# Diabetes Technologies in Ultra-Endurance Type 1 Diabetes: Qualitative Study

**DOI:** 10.2196/86815

**Published:** 2026-05-08

**Authors:** Jean-Charles Vauthier, Lucie Choley, Delphine Arduini, Patrick Mas, Bernard Kabuth

**Affiliations:** 1 Faculty of Medicine, Maieutics and Health Professions, Université de Lorraine, 9 avenue de la Forêt de Haye, BP 20199, Vandoeuvre Lès Nancy, 54505, France, 33 0687701621; 2 Laboratoire INTERPSY EA 4432, Université de Lorraine, Nancy, France; 3 Independent Patient Partner, Saint Restitut, France; 4 Independent Patient Partner, Aigues Mortes, France; 5 Centre Psychothérapique de Nancy (CPN), Université de Lorraine, LAXOU, France

**Keywords:** diabetes mellitus, type 1, type 1 diabetes, continuous glucose monitoring, exercise, self-management, patient participation, qualitative research

## Abstract

**Background:**

Diabetes technologies—including continuous glucose monitoring (CGM), insulin pumps, and hybrid closed-loop systems—have profoundly transformed self-management in type 1 diabetes (T1D). While these technologies offer improved glycemic control and safety, their use in ultraendurance sports introduces specific cognitive, material, and organizational challenges that remain underexplored in digital health research.

**Objective:**

This study aimed to explore how adults living with T1D experience and use diabetes technologies in ultraendurance sports, with particular attention to tensions between autonomy, mental load, and vulnerability.

**Methods:**

We conducted semistructured interviews with 13 French-speaking adults with T1D who had completed at least one marathon or ultra-endurance event within the last 5 years and used ≥1 diabetes technology (CGM, pump, or hybrid closed loop). We adopted constructivist grounded theory (Charmaz), using iterative cycles of line-by-line and focused coding, constant comparison, and memo-writing to build and refine analytic categories. Sampling combined purposive strategies through associations and online communities with theoretical orientation (additional participants sought to elaborate emergent categories). Data collection ceased upon theoretical sufficiency, when further interviews no longer yielded substantively new insights for core categories. Two patient partners contributed to question framing, interim sense-checking, and manuscript review. Reporting followed the COREQ (Consolidated Criteria for Reporting Qualitative Research) checklist.

**Results:**

Five interrelated categories described how athletes negotiated technology in practice: (1) From episodic control to continuous anticipation (reframing glucose management through real-time visibility); (2) Gains in safety and performance (perceived benefits and expanded possibilities); (3) Redistributed mental work (hyper-vigilance, logistics, device management); (4) Keeping things working when they break (fragility in extreme conditions, redundancy, improvisation, and experiential expertise); and (5) Making diabetes visible (technologies mediating identity, solidarity, and stigma). Across categories, participants articulated a tension between optimization-oriented performance and a user-constructed robustness—the capacity to maintain function under uncertainty through redundancy and adaptive know-how.

**Conclusions:**

In ultraendurance contexts, diabetes technologies act as both enablers and obligations: they open participation while shifting and sometimes intensifying cognitive and organizational work. A grounded account centered on robustness-in-use highlights practical implications for clinicians (pre-event routines, redundancy planning), designers (context-aware algorithms; improved physical durability), and policy makers (equitable access and exercise-specific education). These findings underscore the value of constructivist, practice-oriented inquiry to inform digital health tool design and support for people living with chronic illness.

## Introduction

Diabetes technologies—including continuous glucose monitoring (CGM), insulin pumps, and increasingly automated insulin delivery or hybrid closed-loop (AID/HCL) systems—have reshaped self-management in type 1 diabetes (T1D) over the last decade [1]. Beyond well-documented glycemic benefits, these systems have been associated with improvements in quality of life for many users, although the extent and nature of these benefits vary across contexts and populations [1,2]. In ultraendurance sports, where physiological demands and environmental stressors are amplified, such technologies may enable participation but can also introduce distinct challenges in day-to-day decision-making [1,3].

Recent work in exercise physiology and diabetes technology has highlighted how prolonged aerobic activity, heat, altitude, or mechanical stress can alter insulin kinetics, sensor accuracy, and carbohydrate absorption, thereby challenging the promises of algorithmic safety and stability [4,5]. Similar concerns have been raised in endurance athletes without diabetes, where CGM accuracy decreases under movement, heat, and prolonged exertion [6]. Although AID/HCL systems offer more automation than previous generations, they still require substantial user input before, during, and after exercise, including anticipatory basal adjustments, activation of exercise modes, and flexible carbohydrate strategies [4]. These adjustments, critical for preventing hypoglycemia, impose organizational and cognitive work that may be heightened in long-duration events.

Qualitative and patient-centered studies show that diabetes technologies can produce both empowerment and burden: continuous data streams require interpretation, alarms may interrupt activity, and device maintenance generates ongoing logistical work [2,7,8]. Reports of data overload, trust calibration, adhesive failures, and the emotional labor of hyper-vigilance are increasingly documented in real-world settings, suggesting that technological autonomy is contingent and situated rather than inherent to device design [7,8]. These issues may be particularly salient in ultraendurance contexts, where environmental variability and long exposure magnify device fragility.

Recent syntheses emphasize that AID/HCL systems deliver robust gains in time in range and reduce hypoglycemia across age groups and life stages [1], yet they still require substantial user input (eg, carbohydrate announcements) and do not eliminate the need for anticipatory adjustments when exercising. Specifically for exercise, experts recommend pre-emptive basal reductions or activation of exercise modes 60‐120 minutes before activity and additional carbohydrate intake adapted to intensity and duration—practices that impose planning work and may be difficult to implement during long events or unanticipated efforts [4]. These observations point to a persistent gap between algorithmic promises and lived exercise realities [1]. Similar limitations have also been observed in endurance athletes without diabetes, where CGM-informed carbohydrate strategies produced steadier glucose trends but did not improve performance during prolonged exercise [9], suggesting that even CGM-informed strategies may have context-dependent value when demands and exposure accumulate.

From a lived-experience perspective, qualitative and mixed-methods studies highlight both empowerment and cognitive burden associated with CGM and pump-AID/HCL use: constant data streams demand interpretation, alarms can be reassuring yet intrusive, and device maintenance generates logistical work [2,7]. Users report benefits in confidence and reduced diabetes distress [2,5], but also describe anxiety related to device reliability, data overload, or social visibility of devices [7]. The overall picture suggests that technologies may re-distribute rather than erase the mental work of self-management—an effect that prolonged effort, exposure to heat, moisture, or mechanical shocks could intensify in ultra-endurance conditions [1,4,8].

Despite advances in diabetes technologies and growing interest in exercise applications, little is known about how adults with T1D practically adapt these systems in ultra-endurance sports. Existing research has focused primarily on metabolic outcomes, device performance, or short-duration exercise protocols, leaving a gap regarding the situated practices, improvisations, cognitive load, and social negotiations that occur during long-duration efforts. No qualitative study has explored how athletes construct autonomy, robustness, and safety when technologies meet environmental uncertainty. This study addresses this gap by examining how adults with T1D experience and use diabetes technologies in ultra-endurance contexts.

## Methods

### Study Design and Theoretical Orientation

We adopted a constructivist grounded theory (CGT) approach [10], focusing on how meanings and practices are co-constructed between participants and researchers. CGT privileges iterative cycles of data collection and analysis, constant comparison, memo-writing, and category development toward conceptual density rather than hypothesis testing.

### Setting

Participants were adults living with type 1 diabetes (T1D) who train and compete in ultra-endurance disciplines (eg, trail/ultramarathon, triathlon, long-distance cycling). Interviews were conducted between July 4, 2024, and December 19, 2024, via secure videoconference.

### Participants and Recruitment

Eligibility criteria were: age ≥18 years; T1D duration ≥1 year; and completion of ≥1 marathon or ultraendurance event within the past 5 years. We used purposive sampling (maximum variation on age, T1D duration, sport, and technology configuration), complemented by a theoretical orientation of additional contacts to elaborate emerging categories when feasible. Recruitment proceeded through national patient associations, endurance communities, and online groups dedicated to T1D and ultraendurance, as well as limited personal networks. Administrators were contacted when required by platform policies; no group names are reported to preserve community privacy.

In France, the relevant online communities comprise at most ~200 individuals. Of these, ~30 eligible candidates received the study information, ~15 indicated willingness to participate, and 13 were ultimately interviewed. No second recruitment wave was required because interviews rapidly yielded theoretical sufficiency for the developing categories. We note that, according to experienced patient partners and community estimates, the pool of eligible T1D ultraendurance athletes in France is likely <100, which positions our sample as substantively large relative to this niche population. Sex distribution in our interviews mirrored what is commonly observed in endurance racing fields; we address implications of this imbalance in the Limitations section.

To ensure full transparency, we note that exact counts of initial outreach messages were not systematically logged because contact often occurred through community posts and administrator relays. The figures reported above therefore reflect the best available estimates based on administrator analytics and message tracking. This limitation is also acknowledged in the Limitations section.

### Data Collection

Semistructured interviews (47‐68  min) were conducted via secure videoconference between July  4, 2024, and December 19, 2024, audio-recorded with consent and transcribed verbatim. The guide covered athletic and diabetes trajectories; technology configurations and daily/race-day practices; preparation and contingency routines; perceived benefits and constraints; cognitive load; device failures and improvisation; and interactions with clinicians/peers. Field notes and analytic memos were maintained throughout.

### Researcher Positionality and Reflexivity

The lead researcher is a clinician-athlete living with T1D (insider position). This facilitated access and rapport but required sustained reflexivity [11]. We used memo-writing, peer debriefing, and collaborative coding discussions to surface assumptions and interrogate interpretive moves. In line with our constructivist grounded theory stance, we did not compute inter-coder reliability coefficients; meaning was negotiated through iterative discussions and documented decision trails.

### Data Analysis and Coding Information

We followed constructivist grounded theory (Charmaz) with iterative line-by-line initial coding, focused coding, and constant comparison within and across interviews, supported by NVivo (version 1.7.2, Lumivero, 2023). Two researchers (BK and LC) independently coded during the initial round, then met to compare and reconcile codes, refine focused codes, and develop the category structure via memo-supported discussions. Disagreements were resolved through discussion and return-to-data; no third-party adjudication was required. We judged theoretical sufficiency when additional interviewing no longer contributed substantively new insights to our developing core categories (variation, conditions, consequences) [12]. An outline/codebook (labels, brief definitions, representative extracts) and an illustrative coding tree are provided in Multimedia Appendix 1.

### Patient and Public Involvement (PPI)

Two patient partners were engaged before data collection (refining the research question), provided interim feedback on preliminary interpretations, and reviewed the final manuscript for clarity and relevance. Both patient partners also participated as interviewees (role overlap), a decision made to ensure that advisory input remained grounded in first-person experience; they did not participate in coding or editorial decisions. We reflect on potential influences of this overlap in the reflexivity subsection and in the Limitations.

### Ethical Considerations

The study received ethics approval from the South-East VI Research Ethics Committee (Ref: 24.00907.000253). All participants provided written informed consent. Participants did not receive any financial or material compensation for their participation in the study. Data were anonymized and identifying details were removed or altered in excerpts. Reporting follows COREQ (Consolidated Criteria for Reporting Qualitative Research) [13]; the completed checklist is uploaded as a Multimedia Appendix 1.

## Results

### Participant Characteristics and Data Collection

Thirteen adults living with type 1 diabetes (T1D) participated in the study. Participants had diverse ultra-endurance profiles (eg, trail and ultratrail running, marathon running, triathlon, long-distance cycling) and varying durations of T1D and technology configurations. An overview of participant characteristics is provided in Table 1.

**Table 1.** Characteristics of study participants.

ID	Sex	Age	Age at diagnosis	Duration of T1D^a^	Treatment	CGM^b^	HbA1c	Sports practiced	Max distance
S1	M	36	21	15	Pump (Omnipod)	Dexcom	7.8	Ultra-trail, ski, tennis-marathon	200 km
S2	M	26	15	11	MDI^c^ (pens)	Libre 2	N/A^d^	Trail, triathlon, rugby-marathon	80 km
S3	M	53	38	15	Pump (Omnipod Dash)	Dexcom	N/A	Ultratrail-marathon	108 km
S4	M	48	25	23	Pump (Omnipod, Diabeloop planned)	Dexcom G6	7.4	Trail, marathon	59 km
S5	F	49	23	27	Pump	Dexcom	5.8	Trail, ultra cycling, marathon, triathlon	42 km
S6	M	41	19	22	Pump	Dexcom	N/A	Trail-marathon	50 km
S7	M	66	40	26	MDI (pens)	Libre	6.7	Trail-marathon	50 km
S8	M	37	13	24	Pump	Dexcom	N/A	Trail-marathon	60 km
S9	M	46	42	4	MDI (pens)	Libre	N/A	Trail-marathon	42 km
S10	M	38	10	28	Pump	Dexcom	6.4‐6.5	Trail-marathon	100 km
S11	M	44	19	25	Pump	Dexcom	6‐6.2	Trail-marathon	80 km
S12	M	26	23	3	MDI (pens)	Libre	N/A	Trail-marathon	42 km
S13	F	46	16	30	Pump	Dexcom	N/A	Trail-marathon	50 km

a T1D: type 1 diabetes.

b CGM: continous glucose monitor

c MDI: multiple daily injection.

d N/A: not available.

This table summarizes demographic and clinical characteristics of participants, including age, diabetes duration, treatment type, technology use, and athletic background. HbA_1c_ values were not systematically collected; they are reported only when spontaneously mentioned by participants during interviews, in accordance with the protocol approved by the ethics committee.

### Overview of Analytic Categories

Analysis yielded five interrelated analytic categories describing how participants experienced and enacted diabetes technologies in ultra-endurance contexts. These categories capture shifts in glucose management practices, perceived benefits and constraints of technology use, the redistribution of cognitive and logistical work, strategies to cope with technical fragility, and the social and identity implications of visible technologies. Across categories, participants described a persistent tension between performance optimization and robustness-in-use under conditions of uncertainty.

**Figure 1.**
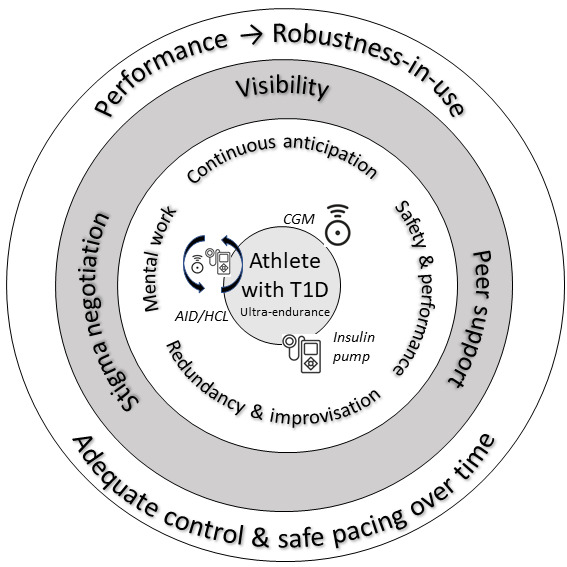
Conceptual map of robustness-in-use in ultra-endurance with T1D. AID/HCL: automated insulin delivery/hybrid closed-loop; CGM: continuous glucose monitoring; T1D: type 1 diabetes.

Figure 1 provides a conceptual overview of how technologies, practice-based strategies, and social dynamics interlock to shape robustness-in-use. This visual summary complements the five analytic categories detailed below.

This sociotechnical figure illustrates how technologies (CGM, insulin pump, AID/HCL), practice-based strategies (continuous anticipation; safety and performance; mental work; redundancy and improvisation), and social dynamics (visibility, peer support, stigma negotiation) interlock to sustain robustness-in-use. Together, these processes extend beyond performance-focused optimization to support “good-enough” control under uncertainty during ultraendurance efforts.

### Analytic Category 1 — From Episodic Control to Continuous Anticipation

Participants described a shift from episodic control—sporadic glucose checks and retrospective corrections—to a mode of continuous anticipation made possible by real-time glucose technologies (CGM, pumps, sometimes AID/HCL). Glucose management became a prospective and dynamic process, in which athletes monitored trends, projected likely trajectories, and adjusted insulin and carbohydrate strategies in advance of anticipated physiological demands.

I see my glucose in real time and I can anticipate things, with the alerts… it’s really convenient.

[S1]

Before, we were practically blind all the time.

[S5]

This anticipatory mode transformed glycemic regulation during ultraendurance efforts. Athletes described constantly looking ahead to the next climb, descent, or aid station, interpreting the CGM curve less as a momentary reading and more as a signal of what would happen minutes or hours later. The shift toward real-time visibility encouraged proactive adjustments—such as pre-emptive basal reductions, timed carbohydrate intake, or temporary targets—often activated before the body’s physiological response became noticeable. For some, this capacity to plan ahead brought a sense of physiological stability and smoother pacing:

The pump was a small revelation for sports… before, I had lots of hypos as soon as I ran 12 or 15 km.

[S8]

However, the degree of anticipation varied with device configuration, terrain, and experience. While CGM alone enabled early warning and forward projection, athletes using pumps—especially hybrid closed-loop systems—reported additional flexibility for fine-tuning insulin delivery. Yet several noted that algorithmic adjustments were “not designed” for prolonged or high-variability exercise, requiring manual overrides or personalized pre-settings. One participant noted:

Control-IQ added mental load… it didn’t know how to manage sport at all

[S13].

Anticipation was especially salient during long climbs, heat exposure, night running, or unpredictable sections of the course, where environmental stressors amplified the need to think ahead and compensate for potential CGM lag, sensor compression, or delayed carbohydrate absorption.

Experience shaped how athletes used data. Some alternated between data-driven projection and embodied sensations, relying on feelings of fatigue, heat, or digestive comfort to interpret CGM signals. Others prioritized bodily cues when technology felt out of sync. Across accounts, continuous anticipation reduced glycemic fluctuations and increased the sense of security but also redistributed cognitive work, requiring ongoing projection, parameter adjustment, and nutritional strategizing in parallel with the physical and navigational demands of ultraendurance effort.

### Analytic Category 2 — Gains in Safety and Performance

Participants described diabetes technologies as enablers of performance, expanding what felt physiologically, medically, and symbolically possible. Devices provided a sense of security, reducing the fear of hypoglycemia, stabilizing glucose trends, and creating conditions that made prolonged or intense effort feel achievable. Several participants associated technology use with a renewed capacity to regulate their bodies during long events, thanks to tools that supported anticipation, stability, and fine-tuned adjustments in real time.

I can reduce my basal rate by 90% one hour before the race… it makes the experience much more comfortable, especially for digestion.

[S3]

For many, performance gains were entangled with a strengthened sense of mastery. Technologies enabled athletes to understand their glycemic patterns more deeply and to act with more precision. The access to continuous data, trend arrows, predictive insights, and adjustable insulin delivery contributed to what several described as a new “physiological literacy”—a clearer, more actionable grasp of how their bodies responded to exertion, terrain, food, and stress.

I understand the kinetics of my glucose levels now.

[S5]

Perceived clinical improvements reinforced this sense of control. Some participants noted reductions in HbA1c, fewer hypoglycemic episodes, or greater time in range, which they interpreted as signs that their training and technological adaptations were working together. These clinical gains were experienced not only as medical improvements but as psychological stabilizers, supporting confidence, self-efficacy, and long-term commitment to ultra-endurance sports.

With the closed-loop system, my HbA1c dropped to 6 or 6.2… I had never gone below 8% before.

[S11]

Technology also enabled participants to challenge external expectations, including medical advice and cultural assumptions about the capabilities of people with T1D. Several described their engagement in ultra-endurance sports as a redefinition of limits, where devices acted as both material and symbolic supports for pushing beyond narratives of fragility or constraint. The ability to complete marathons or ultra-trails was often framed as a form of self-affirmation, emancipation, or identity reconstruction.

They told me I wouldn’t be able to run more than a half marathon… now there are no limits.

[S8]

For some, this expansion of possibility extended beyond sport and influenced their broader life trajectories and relationships. Technologies were woven into stories of belonging, growth, and unexpected positives related to diabetes itself.

Diabetes has brought me so many things… positive things, and lots of meaningful encounters.

[S1]

Across accounts, the perceived benefits of technology were profound but not unconditional. Performance, confidence, and empowerment depended on technical literacy, practical skills, and the capacity to adapt devices to real-world demands. These contingencies foreshadow the subsequent categories, which explore how gains in control coexist with cognitive, material, and emotional burdens, as well as the fragility of technologies under extreme conditions.

### Analytic Category 3 — Redistributed Mental Work

Although diabetes technologies were largely described as empowering, participants also evoked a persistent—and sometimes intensified—mental load associated with their use. The constant availability of glucose data, while reassuring, created a form of hyper-vigilance that extended not only to the body but also to the devices themselves. Athletes emphasized that ultra-endurance sport already requires sustained attention to pace, terrain, nutrition, temperature, and safety; the addition of continuous glucose monitoring layered a parallel stream of cognitive work on top of these existing demands.

Diabetes is present all the time, all the time, all the time…

[S9]

Checking my glucose is the first thing I do when I wake up and the last thing before I sleep.

[S9]

This vigilance did not begin with the race itself but well before it. Preparing for endurance events required significant logistical work, including ensuring that pumps, sensors, adhesive patches, and supplies would last through long distances, unpredictable weather, sweat, or friction. Participants described lengthy and meticulous pre-race routines to secure, test, protect, and calibrate devices. Race-day preparation could be as cognitively taxing as the effort that followed:

You have to think about everything… it takes at least an hour and a half before the race.

[S9]

It’s annoying to change the pump every three days, and to remember everything.

[S7]

Importantly, increased automation did not eliminate this burden. Hybrid closed-loop systems sometimes added new layers of complexity, particularly when algorithmic behavior clashed with the physiological realities of prolonged exertion. Athletes described having to monitor the device’s decisions, override automated actions, or anticipate situations in which the algorithm would not adapt appropriately. Automation, in this sense, did not replace vigilance but redirected it toward the device itself.

Control IQ added mental load… it didn’t know how to manage sport at all.

[S13]

Across accounts, mental load appeared less as an obstacle to performance than as a redistribution of cognitive labor. The work of glycemic management shifted from manual calculations to continuous monitoring, interpretation, and oversight of technological systems. This redistribution could feel worthwhile—because it enabled participation and enhanced safety—but it also demanded sustained attention, layered decision-making, and an acceptance that technology brought its own vulnerabilities. These dynamics connect directly to the next analytic category, which explores how athletes cope when devices fail or underperform during ultra-endurance efforts.

### Analytic Category 4 — Keeping Things Working When They Break

Even as diabetes technologies were described as enabling participation and enhancing control, participants emphasized their material fragility during ultra-endurance events. Long distances, repeated mechanical shocks, perspiration, humidity, transitions in temperature, and the rhythm of movement all contributed to the vulnerability of devices. Pumps, pods, catheter sites, and CGM sensors were depicted as elements that could fail unexpectedly, disrupting both physical effort and the sense of safety cultivated during training.

My catheter got ripped off at the start.

[S7]

Sometimes the pod gets torn off… or the sensor readings don’t match how I feel.

[S9]

These vulnerabilities necessitated a repertoire of redundancy strategies—practices that athletes developed over time to secure devices, anticipate weak points, and prepare for worst-case scenarios. Carrying multiple backup items (pods, catheters, adhesives, batteries, sensors), protecting sites with extra tape or foam, and choosing placement sites less exposed to friction were described as routine elements of pre-race logistics. Redundancy was not optional but part of the craft of running long distances with T1D.

I always take two spare pods.

[S4]

For big races, I bring two extra catheters just in case.

[S11]

When devices failed—whether through accidental detachment, adhesive degradation, CGM drift, or sensor-algorithm mismatch—participants relied on embodied knowledge accumulated over years of living and training with T1D. In the absence of reliable data, they turned to sensations of effort, bodily cues, fatigue patterns, digestive signals, and their understanding of how their glucose typically behaved under similar conditions. This adaptive turn away from technology did not represent a rejection of devices but an assertion of experiential expertise, a reservoir of tacit knowledge that filled technological gaps.

You have to go by feel… it takes years of experience and a certain confidence in yourself.

[S12]

Across accounts, these improvisational skills formed a kind of user-generated robustness. Robustness here did not reside in the devices themselves but in the capacity of athletes to adapt, improvise, and recover control when the technological layer faltered. This required not only planning and technical competence but also emotional steadiness in the face of uncertainty. Device failure could provoke frustration, anxiety, or fear—especially during long races where support is limited—but participants framed resilience as an ongoing process: a combination of preparation, calm evaluation, and embodied know-how.

This category highlights a central paradox of technology in ultra-endurance contexts: the more athletes rely on devices for stability, the more they must prepare for their instability. The work of “keeping things working” is therefore both material and cognitive, intertwining with the mental load described previously and foreshadowing the social and identity dimensions of technology use explored in the next category.

### Analytic Category 5 — Making Diabetes Visible

Participants described the visibility of diabetes technologies—sensors, pumps, adhesive patches, and occasionally association-branded gear—as more than a technical feature: it was a social and symbolic act. Visibility served as a way to assert identity, challenge misconceptions, and embody a stance of openness rather than concealment. Several participants explained that displaying their devices during races or training felt like a form of ownership over the disease, a refusal of shame or silence.

I always said: I’m diabetic and I own it.

[S7]

Another described running as an opportunity to engage others:

It’s about educating people while running.

[S1]

This dimension of visibility was often linked to a sense of responsibility and inspiration. Many participants saw themselves, willingly or not, as role models for younger people with T1D, for families navigating a new diagnosis, or for peers unsure of what was possible. Their presence in ultraendurance events, technologies in plain sight, was meant to broaden assumptions about what living with diabetes allows. For some, the act of being seen was explicitly oriented toward expanding collective horizons:

It’s important to show that the disease doesn’t stop us.

[S5]

We open up possibilities… it reassures parents.

[S8]

Visibility also generated connection. Encounters at races or during long training sessions often led to spontaneous conversations with other runners, spectators, or volunteers who recognized the devices. What might otherwise remain an isolating chronic condition became a channel for recognition, emotional support, and shared experience.

The gear gets noticed… it starts conversations.

[S1]

The community feels like family… it breaks the solitude.

[S2]

Although visibility offered connection and inspiration, participants also described moments of stigma negotiation. Showing one’s devices could draw intrusive questions, misunderstandings, or paternalistic assumptions about fragility. Athletes navigated these encounters by reframing visibility as an act of resistance, turning potentially stigmatizing situations into opportunities for explanation, humor, or quiet defiance. At the same time, visibility created pathways for recognition and belonging: devices sparked spontaneous conversations, generated peer support, and signaled membership in a wider community of endurance athletes living with T1D. Through these interactions, technology became a social interface, contributing to the construction of a shared identity grounded in resilience, capability, and mutual encouragement. In this sense, visibility acted not only as a personal stance but also as a form of collective empowerment, expanding what is seen as possible for people with T1D and reshaping the social meanings of the condition in public athletic spaces.

## Discussion

### Principal Findings

Our findings show how diabetes technologies (CGM, pumps, AID/HCL) reconfigure self-management for adults with T1D engaged in ultra-endurance sports. Beyond glycemic gains, devices reshape the very conditions of participation: they enable anticipatory control (Category 1), unlock perceived performance and confidence (Category 2), redistribute mental work (Category 3), demand user-generated robustness in the face of breakdowns (Category 4), and mediate identity and solidarity (Category 5). Taken together, these categories illuminate the conditional nature of technological autonomy and the situated practices through which athletes keep care working under uncertainty.

### Technological Autonomy Under Conditions

Consistent with recent syntheses on AID/HCL and real-world use, participants described devices as enablers that support tighter regulation and anticipatory adjustments, yet autonomy remained conditional on skills, planning, and context [1,4]. Athletes leveraged real-time data to project forward, pre-tune insulin/carbohydrate strategies, and secure pacing—when devices and environments cooperated. Autonomy, here, was not a default property of technology but the outcome of practice, contingent on technical literacy, race logistics, and environmental stressors. This conditionality reflects broader evidence that AID/HCL systems often struggle in prolonged aerobic exercise, requiring manual overrides, temporary targets, or carbohydrate adjustments to compensate for algorithmic mismatch [4,7].

### Autonomy with a Cost: Redistributed Mental Work

Rather than eliminating workload, technology shifts cognitive demands. Continuous data streams required interpretation and oversight; athletes monitored battery life, adhesion, sensor accuracy, and supply chains, often before the race even started. These observations echo broader evidence that technologies can improve glycemic outcomes and contribute to diabetes distress when use becomes opaque or effortful [2,7]. Automation, especially in AID/HCL, did not erase vigilance; it redirected attention toward the device’s decisions—aligning with the idea that digital systems change the kind of work patients do, not whether work is needed [1]. Similar patterns of redistributed cognitive work have been documented in qualitative studies of CGM and pump users, who describe increased vigilance around alarms, sensor accuracy, device maintenance, and algorithmic decisions, even when automation improves glycemic outcomes [2,5].

### Device Fragility and User-Generated Robustness

Participants framed devices as vulnerable in ultra-settings—detachment, adhesive failure, CGM drift/lag, algorithm–physiology mismatch—calling for redundancy and improvisation. Real-world data and qualitative syntheses likewise note interruptions, inaccuracies, and trust challenges with CGM and pump systems, with glycemic consequences and anxiety when failures occur [7,8]. In our study, robustness did not primarily reside in devices but in users: carrying backups, securing sites, anticipating weak points, and reverting to embodied cues when data became unreliable. These observations are consistent with reports of reduced CGM accuracy under heat and movement and with qualitative accounts in ultra-endurance T1D showing how athletes build robustness through redundancy and on-the-spot improvisation [6,8,14].

### Performance vs Robustness: A Pragmatic Lens, Not a Doctrine

The distinction between performance and robustness helps clarify how athletes actually keep self-management workable under ultra-endurance conditions. From a systems perspective, performance refers to optimization under expected conditions, whereas robustness denotes the ability to maintain function despite variability and perturbation—absorbing errors, adapting, and continuing to operate even when the environment deviates from design assumptions [15].

Our findings suggest that athletes did not pursue perfect optimization during ultra-endurance events. Instead, they enacted a form of robustness-in-use, aiming for “good-enough” stability across long durations, fluctuating terrain, and unpredictable bodily responses. This orientation was visible in anticipatory regulation (Category 1), where real-time visibility and projection enabled athletes to dampen destabilizing swings rather than chase precise targets. It also shaped the performance gains described in Category 2, where confidence and safety were valued because they could hold under shifting conditions, not because they achieved laboratory-level precision.

At the same time, Category 3 (Redistributed Mental Work) showed that robustness required continuous oversight of devices, interpretation of data, and readiness to intervene. Autonomous features of AID/HCL systems supported performance when conditions aligned with algorithmic expectations—but when they did not, robustness depended on the user’s interpretive and corrective capacities. Category 4 (Keeping Things Working When They Break) further illustrated how robustness emerged from redundancy, improvisation, and embodied knowledge: carrying spare equipment, securing adhesives, reverting to capillary checks or bodily cues when CGM data drifted or failed.

Finally, Category 5 (Making Diabetes Visible) highlighted a social dimension of robustness. Encounters with peers, recognition from other athletes, and the ability to reframe stigma supported the emotional steadiness required to troubleshoot under uncertainty. Robustness-in-use was therefore not only technical or cognitive but socially scaffolded, distributed across equipment, skills, and community interactions.

These patterns align with prior work on how digital self-tracking reshapes identity and social positioning and with qualitative accounts in ultra-endurance T1D emphasizing visibility as a form of connection and advocacy [7,14].Taken together, these findings suggest that evaluating diabetes technologies solely on performance metrics (HbA1c, TIR) overlooks a central dimension of real-world functioning: how well systems and users cope with perturbations. Robustness-in-use—anticipation, redundancy, adaptive plasticity, and the relational supports that sustain these practices—was central to autonomy during ultra-endurance, and may be equally relevant in everyday life.

### Lay Expertise in Motion: Situated, Adaptive, Collective

Faced with algorithmic boundaries and environmental unpredictability, participants mobilized lay expertise—trial-and-error learning, embodied sensing, peer exchange—to fine-tune decisions moment by moment. This “practice-based knowledge” aligns with qualitative syntheses showing that users negotiate autonomy through on-the-ground adjustments rather than through stable, standardized routines [2,7]. It also echoes broader digital-health scholarship: self-tracking can foster autonomy through situated negotiation with uncertainty and personal values, not only through control [16]. Consistent with prior qualitative work, including studies in ultra-endurance T1D, such adaptive forms of expertise emerge when digital tools fall short and users must rely on embodied sensing and improvisation [2,14,16]

### Practical Implications

- For clinicians: Translate technology features into exercise-specific routines: pre-emptive basal reductions or exercise modes 60‐120 min before start; timed carbohydrates suited to intensity/duration; explicit redundancy checklists (spare pods/sets, tapes, batteries, backup CGM or capillary tests); scenario planning for adhesion/lag in heat, humidity, or night segments [1,4]. Acknowledge and validate the cognitive work patients do, offering strategies to simplify (eg, standard operating micro-routines, packing templates).
- For designers: Prioritize context-aware algorithms (detecting/proactively handling long aerobic efforts), user-controlled exercise targets that are simple to set and revert, and material durability (adhesives, housings, connectors) for sweat, friction, and temperature swings. Surface transparent device state (eg, adhesion/quality heuristics; lag alerts) to support trust and reduce oversight burden [1].
- For policy and services: Ensure equitable access to advanced systems and to education that explicitly includes endurance-exercise use cases. Support specialized clinics or peer-led workshops where experiential knowledge is shared safely and translated into practical guidance.

### Transferability, Equity, and Diversity of Trajectories

Our sample reflects a male-skewed endurance field and a subset with access to advanced tech, which likely shapes emphasis on vigilance and robustness. Literature in sport/exercise consistently reports under-representation of women, with implications for how needs and design priorities are articulated; services and research agendas should proactively include women and other under-represented groups. Moreover, the “tech-privilege” in our cohort underscores the need to design for graduated complexity and to offer pathways that work for diverse resources, preferences, and bodies [2,7].

### Rethinking Evaluation

These findings argue for evaluation frameworks that complement performance metrics (HbA_1c_, TIR) with robustness-of-use indicators: stability of adhesion, transparency of device state, ease of switching to manual modes, workload to maintain function, preparedness for failure, and user confidence in troubleshooting. In ultra-endurance—an extreme but instructive case—robustness becomes visible; yet the same logic applies to daily life, where uncertainty, variability, and material constraints are the rule rather than the exception.

### Strengths and Limitations

This study presents several strengths. It offers a deep, practice-based understanding of how adults with T1D use diabetes technologies in ultra-endurance contexts—a setting where physiological, environmental, and technological demands converge in distinctive ways. The use of constructivist grounded theory enabled a rich analysis of the processes through which participants enacted anticipation, robustness-in-use, and adaptive expertise. The study also benefited from the involvement of two patient partners, who contributed to the formulation of the research question, provided feedback on emerging interpretations, and reviewed the final manuscript, thereby strengthening the relevance and resonance of the findings.

However, the study also has limitations. The sample reflects the male-skewed composition of ultra-endurance sports, which may influence how certain forms of vigilance, risk management, or identity work are expressed. Access to advanced technologies was common in this cohort, which may limit transferability to athletes with fewer resources, different insurance coverage, or alternative device ecosystems. Recruitment occurred through associations and online communities, and although this strategy enabled access to a niche population, exact counts of initial reach could not be systematically recorded; the reported numbers reflect the best available estimates based on administrator analytics.

Two patient partners also participated as interviewees, creating a role overlap that may have influenced how certain experiences were emphasized. We mitigated this through reflexive memo-writing, peer debriefing, and collaborative coding discussions, emphasizing interpretive transparency rather than inter-coder reliability metrics. Finally, interviews were conducted within a single national context, and ultra-endurance remains a highly selective activity; transferability to everyday physical activity or to broader T1D populations should therefore be considered with caution.

Despite these limitations, the study provides valuable insights into how technological autonomy is enacted under real-world constraints, and highlights dimensions of robustness, adaptation, and experiential knowledge that are relevant beyond ultra-endurance sport.

### Conclusions

Diabetes technologies (CGM, pumps, AID/HCL) are enabling adults with T1D to engage in ultraendurance sports by shifting self-management from episodic control to continuous anticipation and by supporting perceived gains in safety and performance. In real-world practice, however, autonomy remains conditional: technologies redistribute mental work, exhibit material fragilities, and require user-generated robustness through anticipation, redundancy, improvisation, and socially scaffolded support. Rather than a quest for perfect optimization, athletes enact robustness-in-use—keeping care workable across long durations and fluctuating environments. These findings argue for clinical guidance that translates device features into exercise-specific routines, for design priorities that enhance context awareness and material durability, and for evaluation frameworks that complement HbA_1c_/TIR with robustness-of-use indicators (eg, adhesion stability, transparency of device state, ease of switching modes, and troubleshooting confidence). What proves effective in ultraendurance may also inform everyday life with T1D, where uncertainty and variability are the norm.

## Supplementary material

10.2196/86815 Multimedia Appendix 1 A radial coding tree. A central box labeled “Coding tree (T1D ultraendurance)” connects to five analytic categories: Continuous anticipation; Gains in safety & performance; Redistributed mental work; Keeping things working when they break; Making diabetes visible. Each category branches to focused codes, including Projection & anticipation, Stabilization & safety, Logistical & cognitive work, Redundancy & improvisation, Visibility & communication, Stigma & norm negotiation, and Peer & community support.

## Abbreviations

AID/HCL: automated insulin delivery / hybrid closed-loop
CGM: continuous glucose monitoring
CGT: constructivist grounded theory
COREQ: Consolidated Criteria for Reporting Qualitative Research
HbA1c: glycated hemoglobin
ID: participant identifier
MDI: multiple daily injections
NVivo: qualitative data analysis software
PPI: patient and public involvement
T1D: type 1 diabetes
TIR: Time in Range
