# Supplementary figures and images for: Diabetes Technologies in Ultra-Endurance Type 1 Diabetes: Qualitative Study
Source: J Med Internet Res. 2026 May 8;28:e86815. doi: 10.2196/86815 (PMC13154725; doi:10.2196/86815)

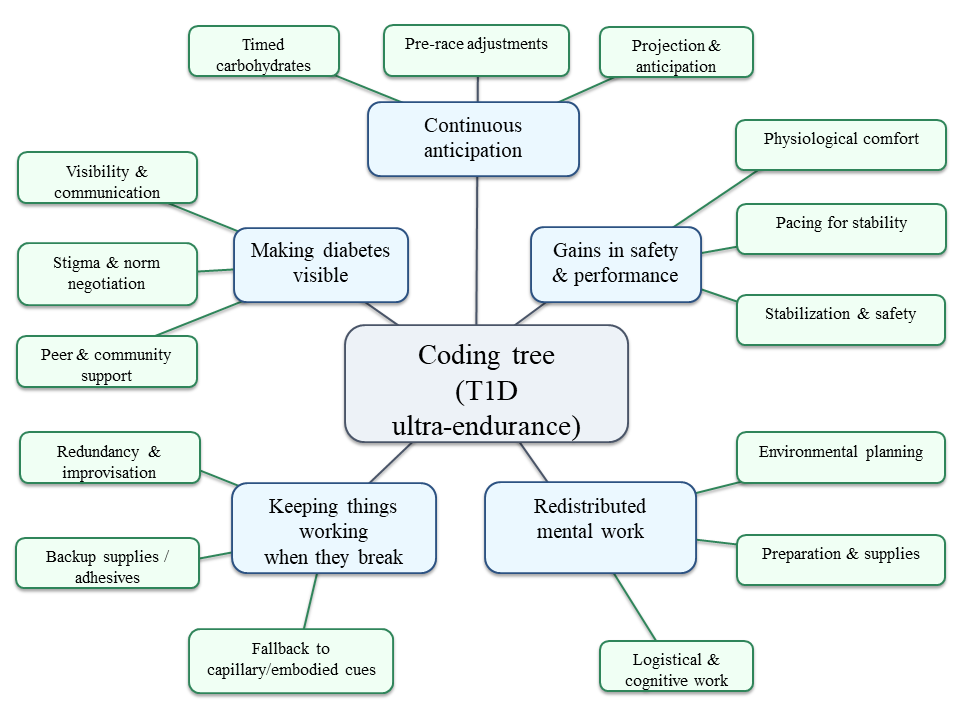

Supplement: Multimedia Appendix 1 [file jmir-v28-e86815-s001.png]
